# Integrated Transcriptomic and Metabolomic Analyses Reveal Diurnal Regulation of Carbon–Nitrogen Metabolism in Maize

**DOI:** 10.3390/genes17060612

**Published:** 2026-05-28

**Authors:** Qingqing Yao, Bin Li, Peiya Wang, Qi Guo, Qiuyan Xiang

**Affiliations:** 1 Key Laboratory of Microbial Resources Exploitation and Application of Gansu Province, Institute of Biology, Gansu Academy of Sciences, Lanzhou 730000, China; libin2753283857@163.com (B.L.); wpeiya@163.com (P.W.); guoqi9207@163.com (Q.G.); 2 Characteristic Microorganisms and Plant Resource Innovation International Scientific and Technological Cooperation Base of Gansu, Institute of Biology, Gansu Academy of Sciences, Lanzhou 730000, China; 3 College of Agronomy and Biotechnology, China Agricultural University, No. 2 Yuanmingyuan West Road, Haidian District, Beijing 100193, China; b20213010057@cau.edu.cn

**Keywords:** maize (*Zea mays* L.), C/N metabolism, diurnal rhythm, transcriptional regulation

## Abstract

Background: Diurnal rhythms are orchestrated by an endogenous clock that synchronizes multiple metabolic processes in plants. While maize growth exhibits diurnal rhythmic characteristics, the regulatory framework governing the rhythmic patterns of its carbon–nitrogen (C/N) metabolism remains to be systematically characterized. Methods: In this study, multi-omics analysis was performed to dissect the diurnal regulatory mechanism responsible for C/N allocation in maize. Results: Maize seedlings display a distinct diurnal pattern in biomass accumulation, characterized by net gains during the light period and partial losses during darkness. A total of 923 metabolites and 3702 rhythmic genes were detected through multi-omics analysis. Among these, 46 rhythmic genes and 16 metabolites were identified within the C/N metabolic pathways, encompassing carbon fixation, the Calvin cycle, starch and sucrose synthesis, glycolysis, the tricarboxylic acid cycle (TCA), the pentose phosphate pathway (PPP), and nitrogen assimilation. Furthermore, *ZmDBB10*, *ZmMYB23*, and *ZmAPRR9* were identified as three candidate transcription factors potentially orchestrating the diurnal characteristics of C/N metabolism. Conclusions: These transcription factors may drive time-dependent metabolite accumulation by modulating key synthase genes within the C/N metabolic network, potentially contributing to diurnal homeostasis of C/N metabolism and promoting growth adaptability in maize.

## 1. Introduction

Maize (*Zea mays* L.) is a globally vital crop for food, feed, and industrial raw materials, and improving its yield is crucial for ensuring food security and promoting sustainable agricultural development [1]. Maize yield largely depends on the coordination and efficiency of carbon (C) and nitrogen (N) metabolism [2]. As two core metabolic processes in plants, C and N metabolism are functionally intertwined and synergistically regulated to co-drive maize growth, development, and essential life activities [3]. A close interaction and balance exist between the two: carbon metabolism supplies energy and carbon skeletons for nitrogen metabolism, while nitrogen metabolism reversely regulates the intensity of carbon metabolism through nitrogen-containing components, thereby helping plants maintain C/N balance under fluctuating environmental conditions [4]. Nitrogen metabolism encompasses the uptake, assimilation, and transport of nitrate and ammonium, as well as the synthesis of nitrogenous compounds such as amino acids, proteins, and nucleic acids [5]. Following root absorption, NO_3_^−^ is sequentially reduced to NH_4_^+^ by nitrate reductase (NR) and nitrite reductase (NiR). Subsequently, glutamine synthetase (GS) catalyzes the condensation of NH_4_^+^ and glutamate (Glu) to generate glutamine (Gln), and then glutamate synthase (GOGAT) transfers the aminoacyl group of Gln to α-ketoglutarate to regenerate Glu [6]. Glu then serves as a precursor for other amino acids via transamination, constituting the material basis of life activities and the source of enzyme systems [7].

Carbon in plants is categorized into two categories: structural carbon (e.g., cellulose, lignin), utilized for morphogenesis, and non-structural carbon (e.g., soluble sugars, starch), which are vital metabolic products that provide a buffering capacity under environmental stress [8,9]. Carbon metabolism initiates with the fixation of CO_2_ through photosynthesis, converting inorganic carbon into organic carbon via enzymatic networks to supply carbon skeletons and energy carriers (ATP, NADPH). This comprehensive process includes the Calvin cycle for triose phosphate synthesis, the synthesis and transport of sucrose and starch, and the synergistic operation of glycolysis, the TCA cycle, and the PPP. Specifically, carbon fixation and the Calvin cycle determine carbon input efficiency, starch and sucrose synthesis govern carbon allocation, glycolysis and the TCA cycle drive energy release, and the PPP provides reducing power and carbon skeletons [10,11]. Plants must maintain a dynamic C/N balance throughout their entire growth period to achieve maximum biomass accumulation, establish and efficiently operate self-built relationships, thereby synergistically improving yield and quality. However, this metabolic network is profoundly restricted by spatiotemporal environment variations [12].

The day-night alternation driven by the Earth’s rotation has prompted plants to evolve a highly conserved endogenous circadian rhythm system [13]. This system integrates internal and external signals to temporally regulate C/N metabolism, serving as a critical hub for maize growth and environmental adaptation. The circadian clock synchronizes the synthesis, transport, allocation, and utilization of photosynthates with diurnal changes [14]. Under light conditions, the rhythmic system modulates photosynthetic gene expression and key enzyme activities within the Calvin cycle, enhancing carbon fixation and photosynthetic assimilation efficiency. During the dark period, the system orchestrates the degradation and mobilization of stored carbohydrates to maintain the balance between respiratory consumption and energy supply [15,16]. Simultaneously, the circadian rhythm coordinates nitrogen metabolism processes such as nitrate absorption, nitrogen assimilation, amino acid synthesis, and protein degradation, ensuring that C/N metabolism is highly synchronized with the diurnal environment [17]. The circadian rhythm-mediated synergistic regulation of C/N metabolism coordinates the allocation and efficient utilization of carbon and nitrogen compounds among different organs, significantly impacting maize growth rate, nutrient uptake and utilization, and biomass accumulation [12,18]. Elucidating this regulatory mechanism helps reveal the inherent laws of crop adaptation to environmental fluctuations, providing a theoretical basis for breeding varieties with high photosynthetic and nitrogen efficiencies.

The circadian rhythm is an endogenous oscillation controlled by the circadian clock system [13]. The classical circadian clock model comprises three components: signal input, a central oscillator, and output pathways [12]. The core oscillator, primarily composed of the morning oscillator genes CCA1 and LHY and the evening oscillator gene TOC1, maintains an intrinsic rhythmic fluctuation with a 24 h period. The clock genes CCA1, LHY, and PRR exhibit peak expression at distinct times of the day and regulate each other. CCA1 and LHY activate PRRs transcription [19], while PRRs conversely provide feedback regulation to CCA1 and LHY [20]. Clock genes maintain their rhythmic expression through multiple negative feedback loops and regulate downstream target genes, affecting the circadian rhythmic growth and development of plants [21,22]. C/N metabolism is finely regulated by the circadian clock system, and MYB family transcription factors LHY and CCA1 regulate C/N metabolism-related genes via their rhythmic expression patterns [14]. CCA1/LHY precisely controls the rate of starch degradation, buffering the massive carbon influx from photosynthesis and ensuring energy supply in the dark [23]. The loss of LHY/CCA1 function diminishes amino acid levels, while PRR7 and PRR9 play opposite roles [24]. Furthermore, Glu and its derivatives regulate CCA1 and affect the expression of nitrogen assimilation genes, indicating a novel mechanism by which nitrogen signals influence circadian clock function [17]. In summary, transcriptional regulation serves as the central link in the interaction between C/N metabolism and circadian rhythms, and a series of core transcription factors serve as “molecular hubs” connecting the rhythmic clock and C/N metabolism during the dynamic responses to light-dark alternation.

The diurnal rhythmicity of C/N metabolism is crucial for optimizing plant carbon and nitrogen utilization and environmental adaptability, but the interaction and transcriptional regulatory mechanisms between C/N metabolism and diurnal rhythms in maize remain unclear. Advances in high-throughput sequencing and multi-omics data integration analysis provides technical support for revealing relevant transcriptional regulatory mechanisms. In this study, seedlings of the maize inbred line ND101 were subjected to continuous 48 h light-dark alternation treatments, integrating transcriptomic and metabolomic analyses to decode the crosstalk between diurnal rhythms and C/N metabolism. By screening rhythmically expressed genes and metabolites, analyzing their expression correlations, and mapping them into a comprehensive metabolic network. Furthermore, Weighted Gene Co-expression Network Analysis (WGCNA) was utilized to identify candidate transcription factors and to construct a rhythmic regulatory network of C/N metabolism with these core transcription factors as hubs. This study aims to clarify the transcriptional mechanisms by which the diurnal rhythms regulate maize C/N metabolism, providing a theoretical basis and molecular targets for breeding maize varieties with efficient C/N utilization and enhanced environmental adaptability.

## 2. Materials and Methods

### 2.1. Plant Materials, Growth Conditions

After disinfection and washing, ND101 seeds were germinated in sand within an incubator under conditions of 28/22 °C, a 14/10 h light-dark cycle, and a relative humidity of 70–80%. Upon emergence and the development of two visible true leaves, the seedlings were transferred to a nutrient solution for cultivation. The nutrient solution contained 0.5 mM MgSO_4_, 0.1 mM KH_2_PO_4_, 1 mM CaCl_2_, 0.1 mM EDTA-Fe, 2 mM KNO_3_, and trace elements (0.03 mM H_3_BO_3_, 0.0025 mM ZnSO_4_, 0.008 mM CuSO_4_, 0.005 mM MnSO_4_, and 0.0003 mM (NH_4_)_6_Mo_7_O_24_) at pH 5.8 [25], and was replaced every 2 days.

### 2.2. Material Collection

Samples were collected at the onset of the light period and the onset of the dark period over two consecutive light-dark cycles. Four maize seedlings with consistent growth were selected. The shoots and roots were separated, and the shoots were placed into sampling bags. The samples were treated at 105 °C for 0.5 h to inactivate enzymes, then dried at 75 °C until constant weight (approximately 3–5 days), and weighed to determine biomass accumulation.

Transcriptomic sampling was performed every 4 h during the first diurnal cycle and every 6 h during the second diurnal cycle. The first cycle employed a 4 h interval to finely resolve rapid changes in gene expression and peak timing, whereas the second cycle used a 6 h interval to maintain full daily cycle coverage while matching the metabolome sampling schedule, thereby facilitating gene–metabolite correlation analysis. A total of 22 RNA sequencing samples were generated from two biological replicates per time point, each replicate consisting of three seedling leaves. Metabolomic sampling was conducted at four time points: light (12 h, 36 h) and dark (20 h, 42 h), with three biological replicates per time point and 3 seedlings per replicate.

### 2.3. RNA Isolation, Transcriptome Sequencing, and Transcriptomic Data Analysis

Total RNA was extracted using TRIzol reagent (Invitrogen, Waltham, MA, USA). RNA library construction followed the standard operational protocol of the kit (Illumina, San Diego, CA, USA), and paired-end sequence reads were obtained on the Nova platform (Illumina). Hisat2-2.0.4, run with default parameters, was employed to align the Illumina sequencing reads to the B73 reference genome (RefGen_v4); the reference genome sequence was derived from the public database (http://ensembl.gramene.org/Zea_mays/Info/Index) [26,27]. The .bam files of uniquely mapped reads were input into the featureCounts (V2.2.0) software, and gene expression levels were calculated based on FPKM values [28].

Circadian rhythm genes exhibiting a 24 h oscillation were identified in the transcriptome data using Molecular timetable and JTK_CYCLE algorithms with high-confidence thresholds (Molecular timetable: CV ≥ 0.3 and R ≥ 0.9; JTK_CYCLE: BH.Q < 0.01). For rhythmicity detection, the JTK_CYCLE algorithm was applied with a false discovery rate (FDR) < 0.05 after Benjamini–Hochberg (BH) correction, a threshold recognized as an optimal balance between controlling FDR and maximizing statistical power. Additionally, the molecular timetable method was employed with a CV ≥ 0.3 to filter for genes with relatively high amplitude, thereby ensuring the robustness of subsequent analyses. The cosine fitting parameters were estimated using the JTK_CYCLE algorithm, in which the period was fixed at 24 h, and the phase represents the time point at which the gene expression peak occurs [29,30]. Based on FPKM values, cosine curve fitting was performed on the expression profile of each gene to screen for circadian genes. Principal Component Analysis (PCA) was performed on all 22 samples using the prcomp function in R software (version 4.3.1, R Core Team, 2013) to evaluate overall correlations. Hierarchical clustering analysis was conducted using gene expression values normalized by log_2_(FPKM + 1). WGCNA was performed using the R package WGCNA for network construction, and the Pearson correlation coefficient cutoff was defined as ≥0.8 [31]. The co-expression network was visualized using Cytoscape software (version 3.8.2). Heatmaps of gene expression patterns were generated using TBtools software (version 2.147) [32]. Gene Ontology (GO) was completed on the online platform eggNOG-mapper (http://eggnog-mapper.embl.de/), and the enrichment of gene functional categories was evaluated based on the annotation results [33].

### 2.4. Metabolites Measurements, Metabolite Mining

Samples were soaked in 70% methanol overnight to extract metabolites. Mass spectrometry data acquisition was performed using Ultra-Performance Liquid Chromatography (UPLC, SHIMADZU Nexera X2, https://www.shimadzu.com.cn/) and Tandem Mass Spectrometry (MS/MS, Applied Biosystems 4500 QTRAP, http://www.appliedbiosystems.com.cn/). Metabolite quantification was analyzed utilizing the multiple reaction monitoring (MRM) mode of triple quadrupole mass spectrometry; Analyst 1.6.3 software was used to parse the mass spectrometry data and obtain the total ion chromatograms and MRM detection results. Metabolite identification was completed based on secondary mass spectrometry alignments against the self-built MWDB database. MultiQuant software was used to obtain the integrated peak area data, where the chromatographic peak area represents the relative content of the corresponding metabolite. PCA and clustering analysis were conducted using R software (http://www.r-project.org/). Pathway enrichment analysis was based on the Kyoto Encyclopedia of Genes and Genomes (KEGG) database, and a threshold of *p* < 0.05 after false discovery rate (FDR) correction (Benjamini–Hochberg method) was considered statistically significant.

### 2.5. Statistical Analysis

Data compilation and graph plotting were performed using Microsoft Excel 2019. Statistical analysis of the data was conducted using SPSS Statistics 26.0 (SPSS Inc., Chicago, IL, USA) via one-way analysis of variance combined with LSD and Duncan’s multiple range test (*p* < 0.05).

## 3. Results

### 3.1. Diurnal Oscillation of Biomass Accumulation

**Figure 1:**
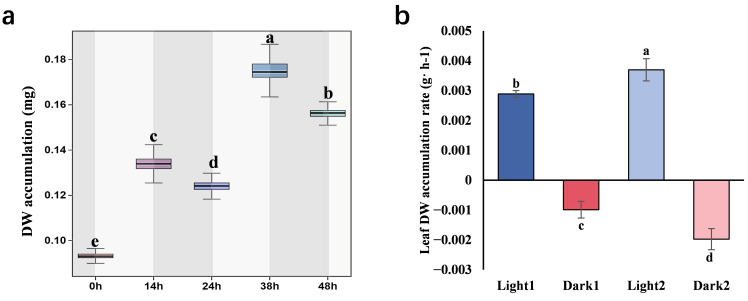
Statistical analysis of shoot biomass in ND101 (WT) seedlings. (**a**) Characteristics of shoot biomass accumulation in ND101 seedlings. Data were presented as mean ± SD (*n* = 4). (**b**) Shoot biomass accumulation rate of ND101 seedlings over two consecutive light-dark cycles. Data were presented as mean ± SD (*n* = 4).

Throughout the growth of the maize seedlings, the biomass accumulation of ND101 seedlings showed an overall increasing trend but exhibited obvious diurnal fluctuations: net biomass increased during the light period and was partially consumed during the dark period, presenting an overall rhythmic variation of “increase-decrease-increase-decrease” (Figure 1a). The biomass accumulation rate was positive during the light period, being significantly higher in the Light2 stage compared to the Light1 stage; the accumulation rate was negative during the dark period, with the consumption rate showing a Dark2 > Dark1 pattern. Both the accumulation rate and the consumption rate increased synchronously as growth progressed (Figure 1b).

### 3.2. Identification of Key Metabolic Pathways Regulating Diurnal Rhythmic Growth

**Figure 2:**
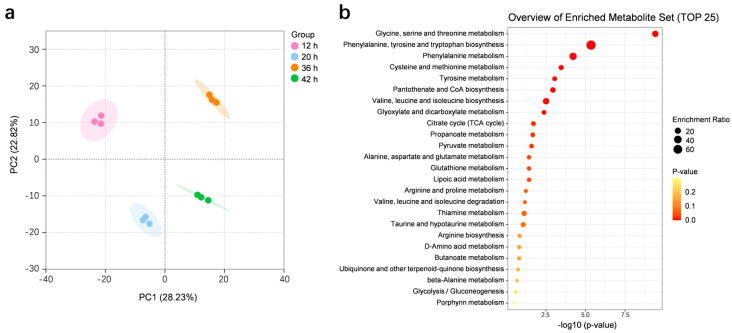
PCA and pathway analysis of metabolomic data. (**a**) PCA revealed 4 distinct groups based on time points in samples collected from ND101 seedlings: 12 h (pink), 20 h (blue), 36 h (orange), and 42 h (green). (**b**) KEGG enrichment analysis was performed for all metabolites in ND101 seedlings. The color of each dot represents the *p*-value, and the size of each dot represents the number of functional metabolites.

To investigate the key metabolic pathways regulating the diurnal rhythmic growth of maize, untargeted metabolomics technology was employed to profile the metabolic profiles of ND101 seedlings at two light time points (12 h, 36 h) and two dark time points (20 h, 42 h). A total of 923 metabolites were detected. PCA of the metabolomics dataset indicated that the metabolomic data at different time points were uniformly distributed according to light (12 h, 36 h) and dark (20 h, 42 h) conditions, demonstrating good reproducibility and inter-group differences (Figure 2a). KEGG pathway enrichment analysis revealed that the detected metabolites were predominantly enriched in amino acid metabolic pathways such as “glycine, serine and threonine metabolism”, “phenylalanine, tyrosine and tryptophan biosynthesis”, and “phenylalanine metabolism” (Figure 2b). These pathways function as fundamental metabolic links, integrating and coordinating carbon and nitrogen metabolism. Furthermore, pivotal carbon metabolism pathways such as “Glyoxylate and dicarboxylate metabolism” and “Citrate cycle (TCA cycle)” were also enriched.

### 3.3. Temporal Expression Profiles and Functional Characterization of Diurnal Rhythmic Genes

**Figure 3:**
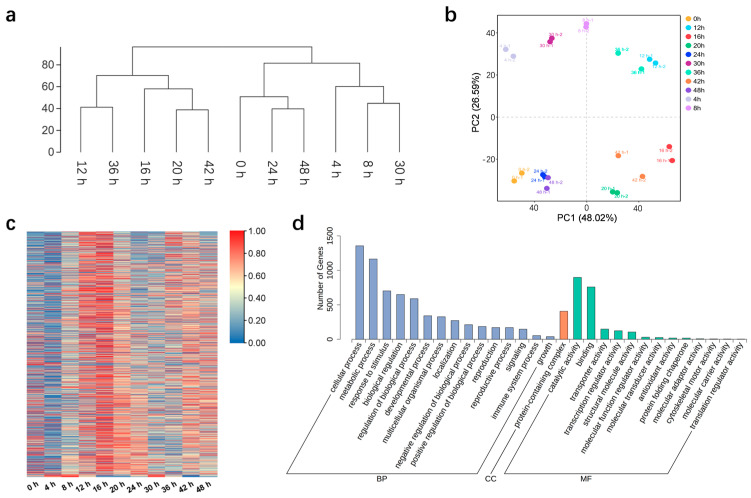
Analysis of clustering, expression profiles, and functional categories of rhythmic genes in ND101 seedlings. (**a**) Hierarchical clustering dendrogram of ND101 seedling samples collected at 11 time points. (**b**) PCA revealed that the 22 samples collected from ND101 seedlings were divided into four distinct groups based on time points. (**c**) Heat map of the expression profiles of 3702 rhythmic genes. The heat map is based on the Z-score (FPKM) values of each gene at 11 time points from ZT0 to ZT48. (**d**) GO functional categories enriched in 3702 rhythmic genes. GO categories were divided into three main groups: CC, MF, and BP.

To elucidate the molecular mechanism regulating maize diurnal rhythmic growth, transcriptome sequencing and data analysis were performed on 22 samples collected across two light-dark cycles. By integrating two rhythmic gene recognition algorithms, 3182 rhythmic genes were screened via the JTK_CYCLE algorithm, and 2244 rhythmic genes were identified using the Molecular Timetable method. A total of 3702 genes exhibiting diurnal rhythmic expression patterns were obtained from the union of the two datasets in ND101 material, including previously reported rhythmic genes such as the classic circadian genes *ZmPRR59*, *ZmTOC2* and *ZmAPRR9* (Figure S1a–c). Cluster analysis of these genes revealed clear time-dependent grouping across the sampled time points (Figure 3a), categorised into four distinct phases: Phase I (0 h, 24 h and 48 h, corresponding to dark periods); Phase II (4 h, 8 h and 30 h, representing light periods); Phase III (12 h and 36 h, light periods); and Phase IV (16 h, 20 h and 42 h, dark periods). The PCA of the transcriptome data presented an obvious separation trend along the time axis (Figure 3b): light period samples (4 h, 8 h, 12 h, 30 h, 36 h) were distributed in the upper part of the PCA plot, and dark period samples (0 h, 16 h, 20 h, 24 h, 42 h, 48 h) were in the lower part; samples close to the light period (0 h, 4 h, 8 h, 24 h, 30 h, 48 h) were skewed to the left, while samples close to the dark period (12 h, 16 h, 20 h, 36 h, 42 h, 48 h) concentrated on the right. This spatial distribution pattern reveals that maize possesses diurnal rhythmic growth characteristics within a 48 h cycle. Analysis of the expression profiles of the 3702 rhythmic genes showed a complete oscillatory cycle over a 24 h period (Figure 3c). The overall expression pattern showed dynamic changes from blue to red and back to blue, or from red to blue and back to red, reflecting the periodic regulatory features of gene expression.

GO annotation categorized the genes into Biological Process (BP), Cellular Component (CC), and Molecular Function (MF) (Figure 3d). Within the BP category, the functional annotations with the largest gene count were “cellular process” and “metabolic process”, indicating that rhythmic genes are fundamentally involved in basic cellular activities and material synthesis and degradation. A subset of genes was also annotated to “response to stimulus”, “biological regulation”, “negative regulation”, and “positive regulation”, revealing that rhythmic genes also play important roles in adaptive responses and precise regulation processes. Other functional annotations had fewer genes, mainly including reproductive and immune system processes. The CC category was primarily annotated as “protein-containing complex”, describing functional structural units assembled from multiple protein subunits through non-covalent interactions. In the MF category, the function with the most annotated genes was “catalytic activity”, demonstrating that a substantial portion of rhythmic genes directly catalyze biochemical reactions to drive complex metabolic and cellular processes. The second most abundant annotation was “binding”, which involves the specific recognition and reversible association of proteins, nucleic acids, small molecules, and ions, forming the basis for molecular interactions, complex assembly, signal recognition, and transcriptional regulation. In addition, a minority of genes were annotated to functions such as “transporter activity”, “transcription regulator activity”, and “structural molecule activity”.

### 3.4. Identification and Correlation Analysis of C/N Metabolic Rhythmic Genes and Metabolites

**Figure 4:**
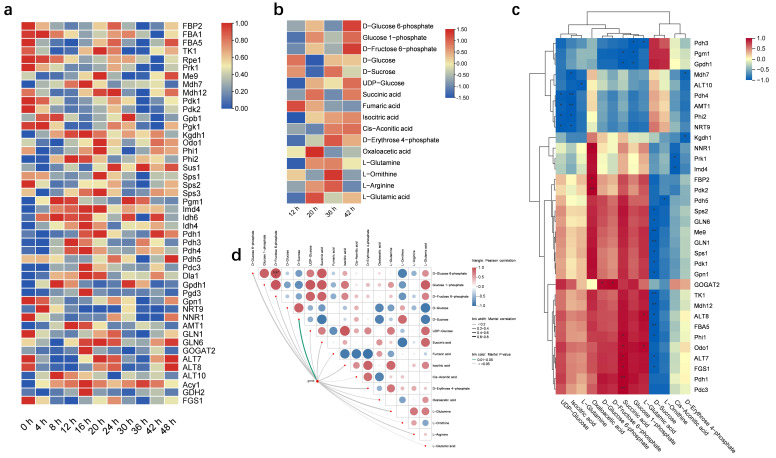
Expression profiling and correlation analysis of metabolites and rhythmic genes in C/N metabolic pathways. (**a**) Expression heatmap of 46 rhythmic genes involved in the C/N metabolic pathway. The heat map is based on the Z-score (FPKM) values of each gene at 11 time points from ZT0 to ZT48. (**b**) Heat map of 16 metabolites in C/N metabolic pathways. (**c**) Correlation analysis of rhythmic genes and metabolites in carbon–nitrogen metabolic pathways. Red indicates a positive correlation, blue indicates a negative correlation. **: *p* < 0.01; *: *p* < 0.05. (**d**) Mantel test heatmap of metabolite-rhythmic gene correlations in C/N metabolic pathways: Red indicates a positive correlation, and blue indicates a negative correlation. **: *p* < 0.01; *: *p* < 0.05. The values within the cells represent the correlation coefficients, and the asterisks indicate significance. The connections in the lower-left corner represent the Mantel test results between metabolites and rhythmic genes. The thickness of the connections represents the overall correlation coefficient.

By analyzing genes enriched in C/N metabolic pathways within the transcriptome data, 46 rhythmic genes were identified (Figure 4a). Among these, 34 were linked to carbon metabolism, predominantly representing families such as MDHs, PDKs, PHIs, SPSs, and LDHs. The remaining 12 were associated with nitrogen metabolism, including nitrate and ammonium transporter genes (*ZmNRT9*, *ZmAMT1*), glutamine synthetase genes (*ZmGLN1*, *ZmGLN6*), alanine aminotransferase genes (*ZmALT7*, *ZmALT8*, *ZmALT10*), as well as *ZmNNR1*, *ZmACY1*, *ZmGDH2*, and *ZmFGS1*. Within the metabolome, 16 metabolites associated with C/N metabolic pathways were identified (Figure 4b), including sugars and sugar phosphates (D-Glucose 6-phosphate, Glucose-1-phosphate, D-Fructose 6-Phosphate, D-Glucose, D-Sucrose, UDP-Glucose), intermediate products of the TCA cycle (Succinic acid, Fumaric acid, Isocitric Acid, Cis-Aconitic acid, Oxaloacetic acid), intermediate products of the PPP (D-Erythrose-4-phosphate), and nitrogen metabolic pathway-related metabolites (L-Glutamine, L-Ornithine, L-Arginine, L-Glutamic acid). Among these 16 metabolites, 12 showed diurnal differences in accumulation: 4 metabolites (D-Glucose, D-Sucrose, Fumaric acid, L-Ornithine) displayed higher accumulation during the light period than the dark period; the remaining 8 metabolites (D-Glucose 6-phosphate, Glucose-1-phosphate, D-Fructose 6-Phosphate, Succinic acid, Isocitric Acid, Oxaloacetic acid, L-Glutamic acid, UDP-Glucose) showed lower accumulation during the light period than the dark period.

Expression correlation analysis was performed on the 46 rhythmic genes and 16 metabolites identified in the C/N metabolic pathways. The multi-omics expression correlation clustering heatmap revealed that 34 genes were correlated with corresponding metabolites, and 12 metabolites were correlated with corresponding rhythmic genes (Figure 4c). Specifically, Sucrose was significantly negatively correlated with the expression of 14 genes; Succinic acid was significantly positively correlated with 6 genes and negatively correlated with 2 genes; Isocitric acid was negatively correlated with 5 genes; and UDP-Glucose was negatively correlated with 4 genes. The Mantel test heatmap results for rhythmic genes and metabolites in C/N metabolic pathways demonstrated that sucrose had the highest correlation coefficient and the most significant correlation with rhythmic genes, revealing that sucrose is a critical node connecting photosynthesis and carbohydrate metabolism (Figure 4d). Furthermore, D-Glucose 6-phosphate, D-Erythrose-4-phosphate, and L-Glutamine also exhibited high correlation coefficients with rhythmic genes, serving as core intermediate products in glycolysis, the PPP, and nitrogen metabolic pathways, respectively.

**Figure 5:**
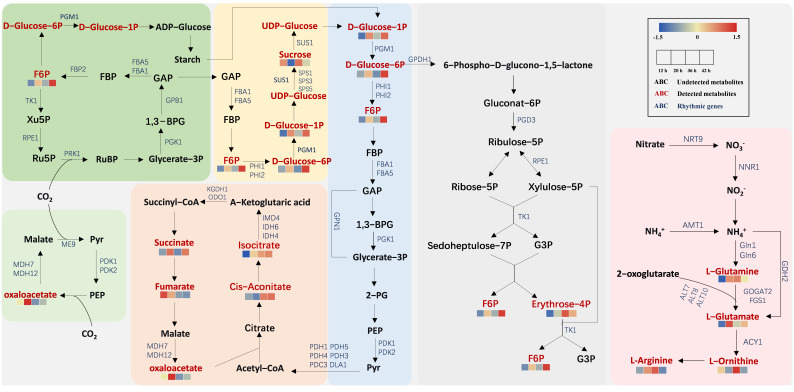
Comprehensive analysis of 16 metabolites and 46 rhythmic genes in C/N metabolic pathways.

A comprehensive analysis of the 16 metabolites and 46 rhythmic genes in C/N metabolic pathways showed that they collectively involve core C/N metabolic pathways, including carbon fixation, the Calvin cycle, starch synthesis, sucrose synthesis, glycolysis, the TCA cycle, the PPP, and nitrogen assimilation (Figure 5). Metabolites in the carbon fixation pathway included oxaloacetate, and associated rhythmic genes included *ZmPDK1/2*, *ZmME9*, *and ZmMDH7/12*. Calvin cycle pathway metabolites included F6P, with genes *ZmTK1*, *ZmRPE1*, *ZmPRK1*, *ZmPGK1*, *ZmGPB1*, *ZmFBA1/5*, and *ZmFBP2*. Starch synthesis pathway metabolites were D-Glucose-6P and D-Glucose-1P, and the gene was *Zm*PGM1. Sucrose synthesis pathway metabolites included F6P, D-Glucose-6P, D-Glucose-1P, UDP-Glucose, and Sucrose, associated with genes *ZmFBA1/5*, *ZmPHI1/2*, *ZmPGM1*, *ZmSPS1/2/3*, and *ZmSUS1*. Glycolysis metabolic pathway involves metabolites F6P, D-Glucose-6P, and D-Glucose-1P, along with genes *ZmFBA1/5*, *ZmPHI1/2*, *ZmPGM1*, *ZmGPN1*, *ZmPGK1*, and *ZmPDK1/2*. The TCA cycle pathway contains metabolites Cis-Aconitate, Isocitrate, Succinate, Fumarate, and oxaloacetate, related to genes *ZmPDH1/3/4/5*, *ZmPDC3*, *ZmDLA1*, *ZmIMD4*, *ZmIDH4/6*, *ZmKGDH1*, *ZmODO1*, and *ZmMDH7/12*. PPP metabolites included Erythrose-4P and F6P, with genes *Zm*GPDH1, *Zm*PGD3, *Zm*RPE1, and *Zm*TK1. The nitrogen metabolism pathway includes metabolites L-glutamine, 2-oxoglutarate, L-glutamate, L-ornithine, and L-arginine, associated with rhythmic genes *ZmNRT9*, *ZmNNR1*, *ZmAMT1*, *ZmGLN1*, *ZmGLN6*, *ZmALT7*, *ZmALT8*, *ZmALT10*, *ZmGOGAT2*, *ZmGDS1*, *ZmGDH2*, and *ZmACY1*.

### 3.5. Identification of Candidate Transcriptional Regulators in Diurnal C/N Metabolism

**Figure 6:**
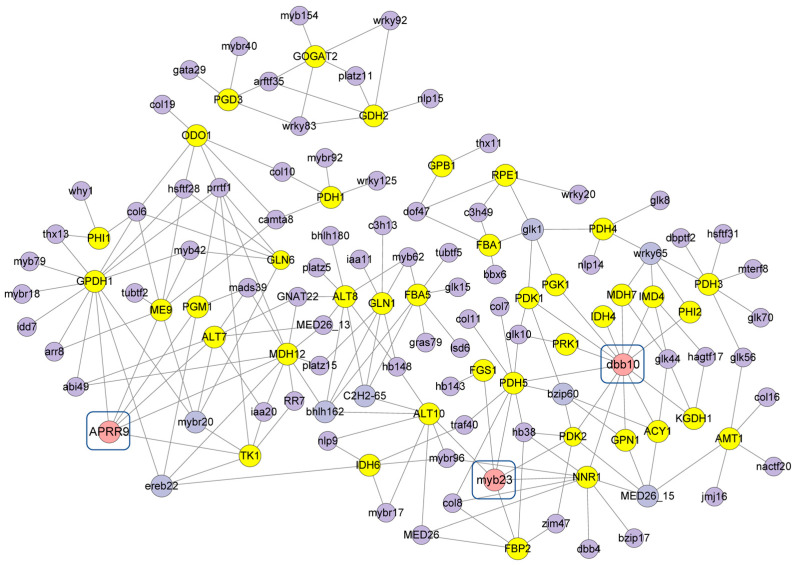
Co-expression network diagram of rhythmic genes and transcription factors in C/N metabolic pathways. Yellow indicates downstream target genes, purple indicates rhythmically expressed transcription factors, and red indicates high-frequency transcription factors.

To investigate the expression correlation of rhythmic genes in the C/N metabolic pathway, co-expression analysis was performed on 39 target genes. Transcription factors with high expression correlation and rhythmic expression patterns were screened, yielding a total of 82 transcription factors (Figure 6). Among them, three transcription factors, *ZmDBB10*, *ZmMYB23*, and *ZmAPRR9,* were found to exhibit diurnal rhythmic expression patterns (Figure S1c,d). These three rhythmic transcription factors showed high-frequency co-expression: *ZmDBB10* was co-expressed with 13 rhythmic genes, *ZmMYB23* and *ZmAPRR9* with 6. There were 12 metabolites showing diurnal accumulation differences in the C/N metabolic pathways: D-Glucose 6-phosphate, Glucose-1-phosphate, D-Fructose 6-Phosphate, D-Glucose, D-Sucrose, UDP-Glucose, Succinic acid, Fumaric acid, Isocitric Acid, Oxaloacetic acid, L-Ornithine, and L-Glutamic acid. Their functionally corresponding rhythmic synthase genes were *ZmSUS1*, *ZmSPS1/2/3*, *ZmPGM1*, *ZmPHI1/2*, *ZmIMD4*, *ZmIDH4/6*, *ZmMDH7/12*, *ZmTK1*, *ZmALT7/8/10*, *ZmGOGAT2*, *ZmFGS1*, *ZmACY1*, and *ZmGDH2*. Among these synthase genes, *ZmPHI2*, *ZmIMD4*, *ZmIDH4*, *ZmMDH7*, and *ZmACY1* were co-expressed with *ZmDBB10*. Specifically, *ZmPHI2* is involved in sucrose synthesis, *ZmIMD4*, *ZmIDH4*, and *ZmMDH7* function in the TCA cycle, and *ZmACY1* participates in nitrogen metabolism. *ZmFGS1* and *ZmALT10* were co-expressed with *ZmMYB23; ZmFGS1* and *ZmALT10* are involved in nitrogen metabolism. *ZmPGM1*, *ZmALT7*, *ZmMDH12*, and *ZmTK1* were co-expressed with *ZmAPRR9*; *ZmALT7* is involved in nitrogen metabolism, *ZmMDH12* in the TCA cycle, and *ZmTK1* in the PPP and RuBP regeneration in the Calvin cycle.

## 4. Discussion

Plants utilize their endogenous circadian clock system to anticipate day-night changes and regulate growth and development to adapt to environmental fluctuations; this mechanism has become a key direction for high-photosynthetic-efficiency breeding in maize [12]. The circadian clock directly affects biomass accumulation efficiency by regulating the balance between carbon fixation in the light and carbon mobilization in the dark [16]. In this study, the biomass accumulation of maize seedlings displayed clear diurnal differences: increasing during the light period and decreasing during the dark period, reflecting diurnal balance variations in C/N metabolism. Photosynthetic production occurs during the light period, presenting a net accumulation of substances, whereas the dark period is dominated by respiratory consumption, mobilizing carbon sources stored during the light period to maintain basal life activities. Our previous study [34] found that leaf elongation possesses a strict diurnal rhythm: the rate during the light period was higher than that in the dark period. The cytological basis is that cell proliferation during the light period was significantly higher than in the dark period, indicating that substances and energy accumulated during the light period are primarily used for cell proliferation and structural carbon construction. Notably, the diurnal rhythm of leaf growth does not exist independently but is co-regulated by the circadian clock and light signals, coupled with carbohydrate supply rhythms [35,36]. Therefore, the circadian clock system precisely regulates maize biomass accumulation and cell growth by integrating light and environmental signals, converting diurnally accumulated substances and energy into diurnal morphological changes in leaves.

The most enriched pathways in the metabolomic data were amino acid metabolism modules, including “glycine, serine and threonine metabolism” and “phenylalanine, tyrosine and tryptophan biosynthesis”. Phenylalanine shows diurnal accumulation differences, provides precursors for secondary metabolism and enhances crop tolerance to stress [37,38]. Fluctuations in free amino acids and primary metabolite contents determine protein synthesis efficiency. Amino acids are products of nitrogen metabolism and raw materials for protein synthesis; the amino acid metabolic module acts as a hub for coordinating C/N metabolism, connecting carbon skeleton supply with nitrogen assimilation utilization [39]. Enrichment analysis also significantly pointed to core carbon metabolic pathways, “glyoxylate and dicarboxylate metabolism” and the “citrate cycle”, proving that the synergistic effect of the C/N metabolic network in diurnal regulation is the core foundation for adaptive plant growth. Four nitrogen metabolic pathway metabolites were identified from the metabolomic data, serving as key carriers for nitrogen assimilation, transport, and redistribution; 12 metabolites were identified in carbon metabolic pathways. Six sugars and sugar phosphates are core components of photosynthate transport, glycolysis, and starch metabolism, and their diurnal accumulation differences reflect the balance between carbon fixation in the light and carbon mobilization in the dark [11]. The TCA cycle encompasses 5 metabolites, whose diurnal accumulation differences directly affect ATP and carbon skeleton supply [40]. D-erythrose-4-phosphate was detected in the PPP, providing pentose phosphates for nucleotide synthesis and generating NADPH [41]. In summary, the diurnal rhythmic growth of maize seedlings is driven by a highly coordinated C/N metabolism network on a temporal scale.

The plant circadian clock regulates the rhythmic expression of a vast number of genes genome-wide, representing the core level of transcriptional regulation, and this feature is highly conserved in maize [42]. In this study, 3702 rhythmic genes showed regular cyclic expression within a 24 h diurnal cycle, verifying the global temporal regulation of gene expression by the endogenous clock. In the BP category among rhythmic genes, the most enriched functions were “cellular process” and “metabolic process”, demonstrating that rhythmic genes are broadly involved in core processes such as cell division, expansion, and substance synthesis and degradation, which is consistent with the observed diurnal fluctuations in biomass and rhythmic cell proliferation in this study. A large number of rhythmic transcription genes identified in maize were enriched in metabolic processes, confirming that the diurnal rhythm regulates plant growth by temporally tuning these fundamental processes [43]. In the MF category, the most annotated term was “catalytic activity”, showing that most rhythmic genes encode metabolic enzymes, driving the diurnal rhythms of C/N metabolism; followed by “binding” and “transcription regulator activity”, which provide a basis for molecular interactions and transcriptional regulation, implying these genes might play rhythmic regulatory roles in specific links. This conclusion is consistent with studies in model plants and crops, confirming that metabolic enzyme genes and transcription factors are core targets of the circadian clock [44,45,46]. In conclusion, rhythmic genes promote the temporal progression of C/N pathways at the metabolic level, regulate cellular processes synergistically for diurnal growth at the cellular level, and achieve functional coordination via transcriptional regulation at the molecular interaction level.

Within C/N metabolic pathways, a total of 46 rhythmic genes and 16 key metabolites were identified. The carbon metabolic pathway contained 34 rhythmic genes, and the nitrogen metabolic pathway contained 12. Of the 16 metabolites, 12 showed significant diurnal accumulation differences: 4 accumulated more during the light period than the dark period, and 8 showed the opposite. Multi-omics correlation analysis and Mantel tests revealed that Sucrose had the highest and most significant correlation coefficient with rhythmic genes. Sucrose can adjust the phase and period of the Arabidopsis circadian oscillator, and the biological clock maintains carbon homeostasis by responding to sucrose signals [47]. In maize, sucrose accumulation may be significantly influenced by diurnally fluctuating environmental conditions; however, the mechanism by which sucrose directly regulates the circadian oscillator in maize has not yet been fully confirmed. Complex associations of succinic acid (positively correlated with 6, negatively with 2), isocitric acid (negatively correlated with 5), and UDP-Glucose (negatively correlated with 4) reflect multi-level regulation between metabolites and enzyme genes in the TCA cycle and sugar metabolic pathways [40]. Moreover, D-Glucose 6-phosphate, D-Erythrose-4-phosphate, and L-Glutamine (core intermediates of glycolysis, PPP, and nitrogen metabolism) also exhibited high correlation coefficients with rhythmic genes, suggesting these metabolic nodes may act as hubs in diurnal rhythm regulation.

Integrating the 16 key metabolites and 46 rhythmic genes into a complete C/N metabolic network reveals multi-layered diurnal regulatory modules. In carbon fixation and the Calvin cycle, oxaloacetate forms a regulatory module with *ZmPDK1/2*, *ZmME9*, and *ZmMDH7/12*; F6P is associated with 7 genes (*ZmTK1*, *ZmRPE1*, *ZmPRK1*, etc.) involved in RuBP regeneration, and its rhythmic fluctuation may dictate diurnal output differences in photosynthates. In the carbohydrate allocation and utilization stage, starch synthesis centers on *ZmPGM1*, which catalyzes the reversible conversion between G6P and G1P, supporting the notion of strict diurnal turnover for starch storage and consumption [48]. The sucrose synthesis pathway involves 5 metabolites, where sucrose synthesis (*ZmFBA1/5*, *ZmPHI1/2*, *ZmSPS1/2/3*, etc.) and glycolysis (*ZmPGK1*, *ZmPDK1/2*, etc.) share metabolites like F6P, G6P, and G1P to synergistically meet dark-period energy demands. Regarding energy and reducing power supply, multiple rhythmic genes in the TCA cycle (*ZmPDH1/3/4/5*, *ZmIDH4/6*, etc.) and 5 metabolites provide carbon skeletons for dark-period energy supply and amino acid synthesis; PPP rhythmic genes (*ZmGPDH1*, *ZmPGD3*, etc.) and metabolites (Erythrose-4P and F6P) coordinate NADPH supply and phenylpropanoid precursor generation. At the C/N intersection, nitrogen absorption, assimilation, and redistribution are tightly regulated by rhythmic genes like *ZmGLN1/6*, *ZmALT7/8/10*, *ZmGOGAT2*, and 4 metabolites. As a TCA intermediate and nitrogen assimilation substrate, 2-oxoglutarate serves as the core node of C/N intersection [49,50].

**Figure 7:**
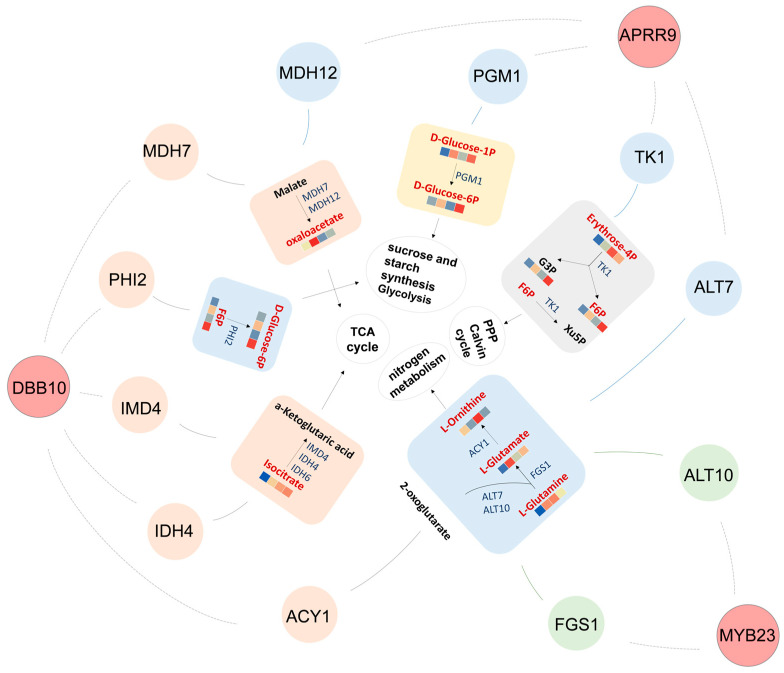
Diurnal regulatory network of carbon–nitrogen metabolism in maize seedlings. *ZmAPRR9*, *ZmDBB10*, and *ZmMYB23* were identified as candidate transcription factors, regulating key enzyme genes involved in the Calvin cycle, starch/sucrose synthesis, glycolysis, the TCA cycle, the PPP, and nitrogen metabolism, thereby affecting metabolite synthesis.

Co-expression network analysis showed that 39 rhythmic genes were co-expressed with 82 transcription factors. *ZmDBB10*, *ZmMYB23*, and *ZmAPRR9* were identified as high-frequency transcription factors, suggesting they may integrate clock and environmental signals, coordinate the time-specific expression of downstream metabolic pathway genes, and potentially play pivotal roles in the C/N metabolism network (Figure 7). B-box (DBB) family proteins can directly integrate light and clock signals to target key metabolic enzyme genes, and their transcription is regulated by circadian rhythms and involved in photomorphogenesis in Arabidopsis. In crops such as rice and maize, the DBB family is associated with key agronomic traits including flowering, secondary metabolism, and yield architecture, serving as strategic targets for the integrated regulation of signaling pathways [51,52,53]. *ZmDBB10* was co-expressed with *ZmPHI2*, *ZmIMD4*, *ZmIDH4*, *ZmMDH7*, and *ZmACY1*, encompassing sucrose synthesis, the TCA cycle, and nitrogen metabolism, suggesting its potential involvement in coordinating carbon flux allocation and nitrogen utilization. MYB transcription factors can regulate carbon skeleton supply and biosynthesis, acting as cores in C/N balance regulation [54,55,56]. As a member of this family, *CCA1* (*ZmCCA1a* and *ZmCCA1b* in maize) temporally activates carbon fixation-related genes, affects photosynthetic efficiency and starch accumulation, and thereby regulates biomass accumulation [57]. *ZmMYB23* was co-expressed with *ZmFGS1* and *ZmALT10*, both of which are involved in nitrogen metabolism, implying that *ZmMYB23* may participate in the regulation of nitrogen assimilation. The clock core component APRR family can bind to metabolic genes and regulate their expression [58,59]. *OsPRR95* and *OsPRR59* affect carbon fixation and sugar output in rice, thereby strengthening the interaction between photosynthetic carbon fixation and the circadian clock [60]. *ZmAPRR9* was co-expressed with *ZmPGM1*, *ZmALT7*, *ZmMDH12*, and *ZmTK1*, targeting the Calvin cycle, starch synthesis, glycolysis, the TCA cycle, the PPP, and nitrogen metabolism, suggesting that it may serve as a key node coordinating the temporal regulation of C/N metabolism across these processes. Therefore, the diurnal accumulation differences in metabolites in C/N metabolic pathways may be associated with the temporal expression of synthase genes regulated by the aforementioned transcription factors.

In this study, diurnal regulatory factors including *ZmDBB10*, *ZmMYB23*, and *ZmAPRR9* were identified through co-expression network analysis, providing candidate genes for dissecting the transcriptional regulatory network of C/N metabolism. As this study represents an exploratory analysis, the regulatory functions of these candidate factors should be further validated through independent experiments and additional biological replicates. Future systematic verification at functional and phenotypic levels will facilitate precise temporal coordination between carbon fixation and nitrogen assimilation. This contributes to enhanced nitrogen use efficiency and photosynthetic performance in maize. The C/N metabolic regulatory network constructed in this study demonstrates clear application potential in maize breeding practices, offering a theoretical basis and genetic resources for developing high-yield and high-efficiency maize germplasm.

## 5. Conclusions

The biomass accumulation and leaf elongation of maize seedlings displayed a significant diurnal pattern. Specifically, a net increase in biomass was observed during the light period, whereas partial biomass consumption occurred during the dark period. By integrating metabolomic and transcriptomic analyses, 3702 rhythmically expressed genes and 923 metabolites were identified, both of which displayed clear temporal clustering patterns. The identified metabolites were mainly enriched in amino acid pathways connecting C/N metabolism and core carbon metabolic pathways, while rhythmic genes were mainly enriched in categories such as “cellular process” and “metabolic process”. Within the C/N metabolic pathways, 46 rhythmic genes and 16 key metabolites were identified, among which 12 metabolites exhibited distinct diurnal accumulation patterns. These metabolites and genes comprehensively cover core biological pathways, including carbon fixation, the Calvin cycle, starch synthesis, sucrose synthesis, glycolysis, the TCA cycle, the PPP, and nitrogen assimilation. Co-expression network analysis elucidated the functional associations of three putative transcription factors (*ZmDBB10*, *ZmMYB23*, and *ZmAPRR9*) and their co-expression modules: *ZmDBB10* may play a role in C/N metabolism, *ZmMYB23* correlates with nitrogen assimilation, and *ZmAPRR9* may participate in integrating photosynthetic carbon reduction, energy metabolism, and nitrogen assimilation. The three candidate transcription factors may synergistically influence the transcription of downstream synthase genes and the diurnal accumulation of metabolites, thereby supporting the diurnal balance of C/N metabolism and the rhythmic growth in maize.

## Data Availability

All summary data were included in the article online at the journal website. Transcriptome information from this research was deposited at the NCBI Sequence Read Archive (http://www.ncbi.nlm.nih.gov/sra (accessed on 24 November 2023)) under accession number PRJNA1044572.

## Supplementary Materials

The following supporting information can be downloaded at: https://www.mdpi.com/article/10.3390/genes17060612/s1, Figure S1: Expression levels (FPKM) of rhythmic genes in RNA-seq date. Respectively, ZmPRR59, ZmTOC2, ZmMYB23, ZmAPRR9, ZmDBB10.

## Abbreviations

The following abbreviations are used in this manuscript:

C/N	Carbon and nitrogen
TCA	Tricarboxylic acid cycle
PPP	The pentose phosphate pathway
GO	Gene Ontology
PCA	Principal Component Analysis
KEGG	Kyoto Encyclopedia of Genes and Genomes
WGCNA	Weighted Gene Co-expression Network Analysis
BP	Biological Process
CC	Cellular Component
MF	Molecular Function
NR	Nitrate reductase
NiR	Nitrite reductase
GS	Glutamine synthetase
Glu	Glutamate
Gln	Glutamine
GOGAT	Glutamate synthase
