# Supplementary material for: Integrated Transcriptomic and Metabolomic Analyses Reveal Diurnal Regulation of Carbon–Nitrogen Metabolism in Maize
Source: Genes (Basel). 2026 May 28;17(6):612. doi: 10.3390/genes17060612 (PMC13299388; doi:10.3390/genes17060612)
Supplement: Supplementary file 1 [file genes-17-00612-s001.zip › genes-4325983-supplementary.pdf]

SUPPORTING INFORMATION

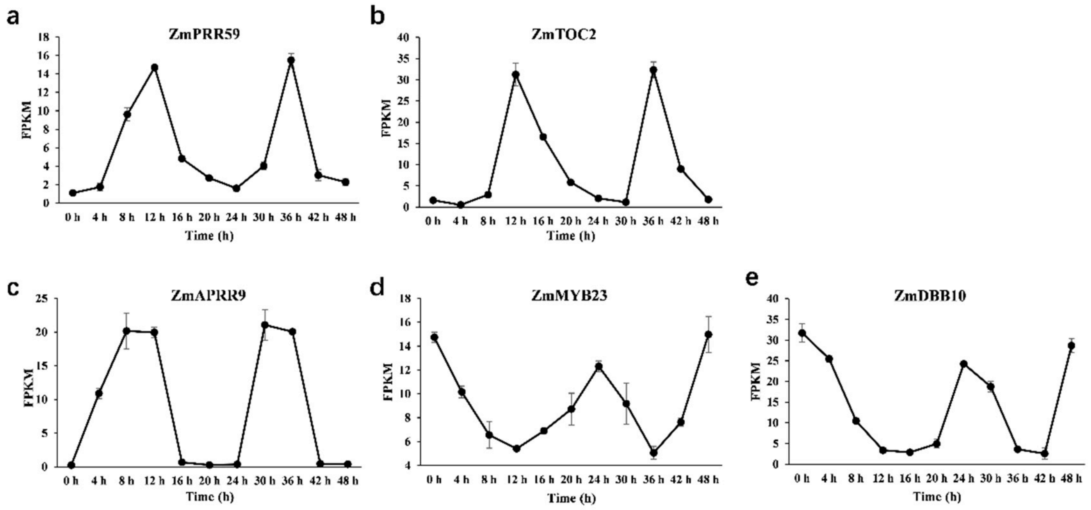

Figure S1. Expression levels (FPKM) of rhythmic genes in RNA-seq date. Respectively, *ZmPRR59*, *ZmTOC2*, *ZmMYB23*, *ZmAPRR9*, *ZmDBB10*.
